# In‐Hospital Mortality and Severe Respiratory and Renal Outcomes—A Territory‐Wide Comparison Between RSV and Influenza

**DOI:** 10.1111/irv.70130

**Published:** 2025-06-20

**Authors:** Wang Chun Kwok, Isaac Sze Him Leung, James Chung Man Ho, Chung Ki Tsui, David Chi Leung Lam, Mary Sau Man Ip, Kelvin Kai Wang To, Desmond Yat Hin Yap

**Affiliations:** ^1^ Division of Respiratory Medicine, Department of Medicine The University of Hong Kong, Queen Mary Hospital Pokfulam Hong Kong SAR; ^2^ Department of Statistics The Chinese University of Hong Kong Shatin New Territories Hong Kong SAR; ^3^ Department of Microbiology, School of Clinical Medicine, Li Ka Shing Faculty of Medicine The University of Hong Kong Pokfulam Hong Kong SAR; ^4^ Division of Nephrology, Department of Medicine The University of Hong Kong, Queen Mary Hospital Pokfulam Hong Kong SAR

**Keywords:** acute kidney injury, chronic kidney disease, dialysis, mortality, respiratory syncytial virus

## Abstract

**Introduction:**

Respiratory syncytial virus (RSV) and influenza virus are important respiratory viruses. Although RSV vaccines have been developed and recommended for patients aged ≥ 60, there is limited data on the clinical impact among the non‐elderly population. It is also important to know the patient subgroups that are at risk of complications from RSV infections.

**Methods:**

We conducted a territory‐wide retrospective study on adults hospitalized for RSV or influenza virus infection between 1/1/2016 and 6/30/2023 in Hong Kong. The in‐patient mortality, severe respiratory failure (SRF), secondary bacterial pneumonia, and acute kidney injury (AKI) were compared. Subgroup analyses were performed in different age groups. The risk factors for mortality and serious respiratory outcomes were assessed.

**Results:**

A total of 41,206 and 3565 patients were hospitalized for influenza and RSV infections. Patients with RSV infection showed a significantly higher risk of in‐patient mortality, SRF, secondary bacterial pneumonia, and AKI compared with those with influenza (*p* < 0.001, for all), and the results were consistent for patients aged ≥ 60, < 60, and 50–59. End‐stage kidney disease requiring real replacement therapy was an independent risk factor for in‐patient mortality and serious respiratory outcomes in RSV infection across different age groups (*p* < 0.001, for all).

**Conclusions:**

Adults hospitalized for RSV infection were associated with a significantly increased risk of in‐patient mortality and adverse respiratory and kidney outcomes than those with influenza. The findings are consistent across various age groups, and the results call for an update on RSV vaccination recommendations in adults, especially for vulnerable subgroups.

## 1 Introduction

Respiratory syncytial virus (RSV) is the third most common respiratory virus causing hospitalization [1, 2, 3]. In adults, RSV can cause a spectrum of lower respiratory tract disease [4]. Severe in‐hospital complications are common among adult patients hospitalized with RSV infections [5] with underlying comorbidities being the risk factors [6]. Earlier reports in Hong Kong suggested that RSV can cause severe respiratory complications in older adults, with prolonged hospitalization, and high mortality similar to seasonal influenza [7]. Despite evidence suggesting RSV may result in more adverse outcomes than influenza, most of the studies had a rather short coverage period, and those with longer coverage periods were limited by their relatively small sample size [8, 9, 10, 11]. To complicate the matter further, the emergence of COVID‐19 may have also altered the usual distribution and outcomes of seasonal respiratory viruses [12, 13, 14, 15].

The mainstay of treatment for RSV infection is supportive therapy [16, 17]. Effective vaccination remains the most important means to prevent severe complications. In Hong Kong, the influenza vaccine was recommended by the Centre for Health Protection in persons aged ≥ 50 [18]. Similar vaccination recommendations for influenza vaccination among the nonelderly population are also adopted in other countries such as South Korea [19], the United States [20], Australia [21], Austria, Poland, and Malta [22]. One should appreciate that vaccination for seasonal influenza and COVID‐19 has been widely promulgated in recent years, but vaccination for RSV is relatively uncommon. Although RSV vaccines have demonstrated acceptable safety and efficacy in adults aged ≥ 60 years [23, 24], the vaccination uptake rates remain low, and many countries do not have clear vaccine recommendations [6]. Whether it shall be offered to patients aged < 60, in particular those with comorbidities, is yet to be determined. There are also financial concerns from a population‐based vaccination program for a newly developed vaccine. Hence, it is crucial to identify at‐risk patients who will develop serious complications when hospitalized for RSV infections, thereby maximizing the cost‐effectiveness of the vaccines. Based on these backgrounds, this territory‐wide study set forth to compare the mortality, respiratory, and kidney outcomes of patients hospitalized for RSV and seasonal influenza among patients from different age groups, including those not yet recommended for RSV vaccination. This study aimed to compare in‐hospital mortality, severe respiratory failure (SRF), secondary bacterial pnuemoniand renal outcomes between RSV and influenza in hospitalized adults, using a territory‐wide retrospective cohort in Hong Kong. The study also assessed the risk factors for adverse clinical outcomes in patients hospitalized for RSV infection. Although the current guidelines only recommend RSV vaccines in patients aged > 60, we also aimed to explore patient groups aged < 60 who are prone to serious complications of RSV infection. Such data will provide evidence to extend RSV vaccination to at‐risk patients who are not yet recommended to receive RSV vaccines.

## 2 Methods

### 2.1 Study Design and Participants

This was a territory‐wide retrospective study to compare first mortality and serious in‐hospital outcomes in adult patients hospitalized for RSV and seasonal influenza infections. We then examined the risk factors for mortality and serious clinical outcomes in adult patients hospitalized for RSV infection in the second session. Adult patients who were admitted to public hospitals in Hong Kong for RSV infection and seasonal influenza between January 1, 2016, and June 30, 2023, were included. Patients were identified from the Clinical Data Analysis and Reporting System (CDARS) of Hospital Authority (HA) by the International Classification of Diseases, Ninth Revision code of 487.8 for seasonal influenza and 079.6 for RSV infections. CDARS is an electronic health record database managed by the HA, which is a public healthcare service provider that has covered > 90% of the Hong Kong population since 1993 [25, 26, 27]. The patients included have laboratory‐confirmed influenza or RSV infections by multiplex polymerase chain reaction (PCR) in respiratory specimens.

### 2.2 Outcome Measurements

The first main exposure of interest was RSV and seasonal influenza. The main outcomes of interest were (1) death during hospitalization, (2) SRF requiring invasive or noninvasive mechanical ventilation (SRF), (3) secondary bacterial pneumonia, and (4) acute kidney injury (AKI). AKI was defined according to the RIFLE criteria [28]. Secondary bacterial pneumonia was defined as the compatible radiological changes on the chest radiograph with supporting laboratory parameters (leukocytosis, neutrophilia) that necessitate systemic antibiotic treatment. We first compared the incidence rates of the above outcomes between patients hospitalized for seasonal influenza and RSV infections.

Next, we explored the risk factors of developing the above outcomes among patients with RSV infections.

### 2.3 Predictive Covariates

The following covariates were assessed as potential risk factors associated with the outcomes: age and Charlson comorbidity index (CCI) as continuous variables; sex; history of malignancy; and underlying DM, chronic airway diseases (asthma, COPD, bronchiectasis), cardiovascular/cerebrovascular diseases (ischemic heart disease, peripheral vascular disease, ischemic stroke), underlying kidney diseases as categorical variables. For underlying kidney diseases, patients were further subclassified into end‐stage kidney disease (ESKD) requiring renal replacement therapy (RRT) (i.e., patients on dialysis or kidney transplantation), patients on dialysis, patients on peritoneal dialysis (PD), patients on hemodialysis (HD), kidney transplant recipients (KTR), and chronic kidney disease (CKD) (defined as estimated glomerular filtration rate [eGFR] < 60 mL/min/1.73 m^2^) [29].

### 2.4 Statistical Analysis

Descriptive tables were created to present the incidence rates of severe in‐hospital outcomes stratified by seasonal influenza and RSV infections, with demographic and clinical data described in actual frequency or mean ± standard deviation (SD), or median (interquartile range [IQR]) where appropriate. Baseline demographic and clinical data were compared between the patients with seasonal influenza and RSV by independent *t*‐test or Mann–Whitney *U* test where appropriate. To assess the risks of severe RSV infections in patients whose age do not fall into the recommendations for RSV vaccination, we performed subgroup analyses for patients aged ≥ 60 or < 60 as well as those aged 50–59. The patients were stratified into these three age groups as they have different recommendations for RSV vaccines. US CDC recommended patients aged ≥ 60 to receive RSV vaccination. The latest recommendation also suggests RSV vaccination to be administered in those aged < 60 but with risks of severe infection. Adjuvanted subunit vaccine is to be used in persons 50–59 years old, and the bivalent vaccine has been approved for persons 18–59 years of age. As such, we stratified the cases into these three groups, which reflect the different subgroups recommended for different RSV vaccines. In order to assess the severity of disease among different age groups with different vaccine recommendations, subgroup analyses were done in these subgroups.

To ensure a more robust comparison on patient outcomes, we also performed propensity score matching (PSM) to match for age, sex, ethnic group, history of malignancy, presence of DM, chronic airway diseases, cardiovascular/cerebrovascular diseases, ESKD requiring RRT, and CCI, which are the potential confounding factors for disease severity, with 1:1 matching and caliper of 0.2 times SD of the logit of propensity score.

To compare the risk of mortality and serious in‐hospital complications between patients hospitalized with seasonal influenza and RSV infections, we first performed univariate logistic regression analyses followed by multivariable analysis. The risk factors for adverse clinical outcomes in patients hospitalized for RSV infection were assessed first by univariate and then multivariable analyses. The covariates adjusted in the multivariable analyses included age, sex, CCI, presence of diabetes mellitus (DM), chronic airway diseases, cardiovascular/cerebrovascular diseases, and previous vaccination against influenza and pneumococcal polysaccharide and conjugated vaccine.

Data analyses were performed using R version 4.0.3 software (R Core Team, Vienna, Austria). For all statistical analyses, statistical significance was assessed at a *p* level of <0.05. STROBE and RECORD reporting guidelines were followed in the generation of this report.

### 2.5 Ethical Considerations

The study was approved by the Institutional Review Board (IRB) of the University of Hong Kong and HA Hong Kong West Cluster (UW 24–137). Patient informed consent was waived in this retrospective study by the IRB as it is a retrospective study without active patient recruitment, whereas the data were already deidentified. The study was conducted in compliance with the Declaration of Helsinki.

## 3 Results

### 3.1 Patients' Characteristics

A total of 41,206 and 3565 adult patients were hospitalized for seasonal influenza and RSV infections in public hospitals in Hong Kong during the period of January 1, 2016, to June 30, 2023 (Table 1 and Figure S1). Patients with RSV infections were older, had a higher proportion of males and other comorbidities than those with seasonal influenza (Table 1). Among the patients hospitalized for influenza, 41,046 received oseltamivir, nine received baloxavir, 51 received peramivir, 100 received zanamivir, and one received rimantadine.

**TABLE 1:** Baseline clinical characteristics for the full cohort and propensity score matched (1:1) cohort.

		All	Whole cohort		*p*	ASD	Propensity score matched cohort		*p*	ASD
			Influenza virus A(H1N1), A(H3N2), and B	RSV			Influenza virus A(H1N1), A(H3N2), and B	RSV		
Number of subjects (*N*)		44,771	41,206	3565			3565	3565		
Age, years (Mean ± SD)		67.9 ± 19.7	67.4 ± 19.8	74.3 ± 16.2	< 0.001*	0.383	74.8 ± 16.2	74.3 ± 16.2	0.209	0.030#
Male, *N* (%)		23,008 (51.4%)	21,036 (51.1%)	1972 (55.3%)	< 0.001*	0.086#	1958 (54.9%)	1972 (55.3%)	0.757	0.008#
Body mass index, kg/m ^2^ (Mean ± SD)		24.4 ± 4.6	24.5 ± 4.6	24.1 ± 4.8	0.06	0.095#	24.4 ± 4.4	24.1 ± 4.8	0.09	0.085#
Ethnicity, *N* (%)	Chinese	43,366 (96.9%)	39,856 (96.7%)	3510 (98.5%)	< 0.001*	0.133	3479 (97.6%)	3510 (98.5%)	0.007*	0.095
	Northeast Asian	23 (0.0%)	21 (0.1%)	2 (0.06%)			1 (0.03%)	2 (0.06%)		
	Southeast Asian	637 (1.4%)	609 (1.5%)	28 (0.8%)			39 (1.1%)	28 (0.8%)		
	South Asian	520 (1.2%)	510 (1.2%)	10 (0.3%)			33 (0.9%)	10 (0.3%)		
	Caucasian	17 (0.0%)	17 (0.0%)	0 (0.0%)			1 (0.03%)	0 (0.0%)		
	Others	208 (0.5%)	193 (0.5%)	15 (0.4%)			12 (0.3%)	15 (0.4%)		
eGFR (Mean ± SD)		64.9 ± 26.4	65.1 ± 26.4	62.5 ± 26.7	< 0.001*	0.097#	64.8 ± 25.4	62.5 ± 26.7	< 0.001*	0.090
ESKD requiring RRT, *N* (%)		1716 (3.8%)	1540 (3.7%)	176 (4.9%)	< 0.001*	0.059#	141 (4.0%)	176 (4.9%)	0.051	0.048#
Renal transplant, *N* (%)		300 (0.7%)	274 (0.7%)	26 (0.7%)	0.730	0.008#	16 (0.4%)	26 (0.7%)	0.164	0.037#
Peritoneal dialysis, *N* (%)		823 (1.8%)	725 (1.8%)	98 (2.7%)	< 0.001*	0.067#	71 (2.0%)	98 (2.7%)	0.043*	0.050#
Hemodialysis, *N* (%)		1145 (2.6%)	1025 (2.5%)	120 (3.4)	0.002*	0.052#	94 (2.6%)	120 (3.4%)	0.083	0.043#
CKD stage, *N* (%)	1	30,079 (67.2%)	27,693 (67.9%)	2386 (66.9%)	< 0.001*	0.105	1911 (53.6%)	2386 (66.9%)	< 0.001	0.317#
	2	7437 (16.6%)	6919 (16.8%)	518 (14.5%)			909 (25.5%)	518 (14.5%)		
	3	5374 (12.0%)	4924 (11.9%)	450 (12.6%)			565 (15.8%)	450 (12.6%)		
	4	1129 (2.5%)	1011 (2.5%)	118 (3.3%)			101 (2.8%)	118 (3.3%)		
	5	752 (1.7%)	659 (1.6%)	93 (2.6%)			79 (2.2%)	93 (2.6%)		
Cardiovascular diseases, *N* (%)		15,450 (34.5%)	13,832 (33.6%)	1618 (45.4%)	< 0.001*	0.244	1614 (45.3%)	1618 (45.4%)	0.943	0.002#
Airway diseases, *N* (%)		7004 (15.6%)	6182 (15.0%)	822 (23.1%)	< 0.001*	0.206	820 (23.0%)	822 (23.1%)	0.978	0.001#
Diabetes mellitus, *N* (%)		11,066 (24.7%)	10,061 (24.4%)	1005 (28.2%)	< 0.001*	0.086#	995 (27.9%)	1005 (28.2%)	0.812	0.006#
Malignancies, *N* (%)		3056 (6.8%)	2780 (6.7%)	276 (7.7%)	0.026*	0.038#	249 (7.0%)	276 (7.7%)	0.238	0.029#
Active solid organ malignancy, *N* (%)		2492	2292 (5.6%)	200 (5.6%)	0.901	0.007#	194 (5.4%)	200 (5.6%)	0.22	0.007#
Past history of malignancy, *N* (%)		88	67 (0.16%)	21 (0.6%)	< 0.001*	0.26	30 (0.8%)	21 (0.6%)	0.22	0.007#
Active lymphoid malignancies, *N* (%)		232	207 (0.5%)	25 (0.7%)	0.113	0.009#	11 (0.3%)	25 (0.7%)	0.016*	0.19
Past history of lymphoid malignancies, *N* (%)		3	1	2 (0.06%)	0.143	0.009#	1 (0.02%)	2 (0.06%)	0.025*	0.15
Active myeloid malignancies, *N* (%)		230	192 (0.5%)	38 (1.1%)	< 0.001*	0.22	12 (0.3%)	38 (1.1%)	< 0.001*	0.34
Past history of myeloid malignancies, *N* (%)		21	21	0 (0%)	< 0.001*	0.24	1 (0.02%)	0 (0%)	< 0.001*	0.23
CCI (Mean ± SD)		3.8 ± 2.5	3.7 ± 2.6 4 (2–6)	4.6 ± 2.3 5 (3–6)	< 0.001*	0.368	4.6 ± 2.3 5 (3–6)	4.6 ± 2.3 5 (3–6)	0.539	0.015#
LAMA, *N* (%)		3939 (8.8)	3465 (8.4)	474 (13.3)	< 0.001*	0.158	403 (11.3)	474 (13.3)	0.012*	0.061#
LABA, *N* (%)		5309 (11.9)	4638 (11.3)	671 (18.8)	< 0.001*	0.213	556 (15.6)	671 (18.8)	< 0.001*	0.086#
ICS, *N* (%)		5047 (11.3)	4484 (10.9)	563 (15.8)	< 0.001*	0.145	555 (15.6)	563 (15.8)	0.820	0.006
Influenza vaccine, *N* (%)		13,795 (30.8%)	12,557 (30.5%)	1238 (34.7%)	< 0.001*	0.091#	1242 (34.8%)	1238 (34.7%)	0.941	0.002#
Pneumococcal conjugated vaccines, *N* (%)		5089 (11.4%)	4698 (11.4%)	391 (11.0%)	0.450	0.014#	436 (12.2%)	391 (11.0%)	0.104	0.039#
Pneumococcal polysaccharide vaccine, *N* (%)		1672 (3.7%)	1541 (3.7%)	131 (3.7%)	0.880	0.003#	140 (3.9%)	131 (3.7%)	0.620	0.013#

Abbreviations: ASD = absolute standardized difference; CCI = Charlson comorbidity index; CKD = chronic kidney disease; eGFR = estimated glomerular filtration rate; ESKD = end‐stage kidney disease; ICS = inhaled corticosteroid; LABA = long‐acting beta‐agonists; LAMA = long‐acting muscarinic antagonists; mmol = millimoles per liter; RRT = renal replacement therapy; SD = standard deviation.

* = statistically significant; # = good balance with ASD < 0.1.

### 3.2 Severe In‐Hospital Outcomes Among Influenza and RSV Patients

#### 3.2.1 Whole Cohort

Adults who were hospitalized for RSV infection had significantly higher rates of mortality (10.1% vs. 5.5%, *p* < 0.001), SRF (22.7% vs. 13.3%, *p* < 0.001), secondary bacterial pneumonia (61.5% vs. 39.5%, *p* < 0.001), and AKI (16.0% vs. 12.6%, *p* < 0.001) compared with seasonal influenza.

**FIGURE 1:**
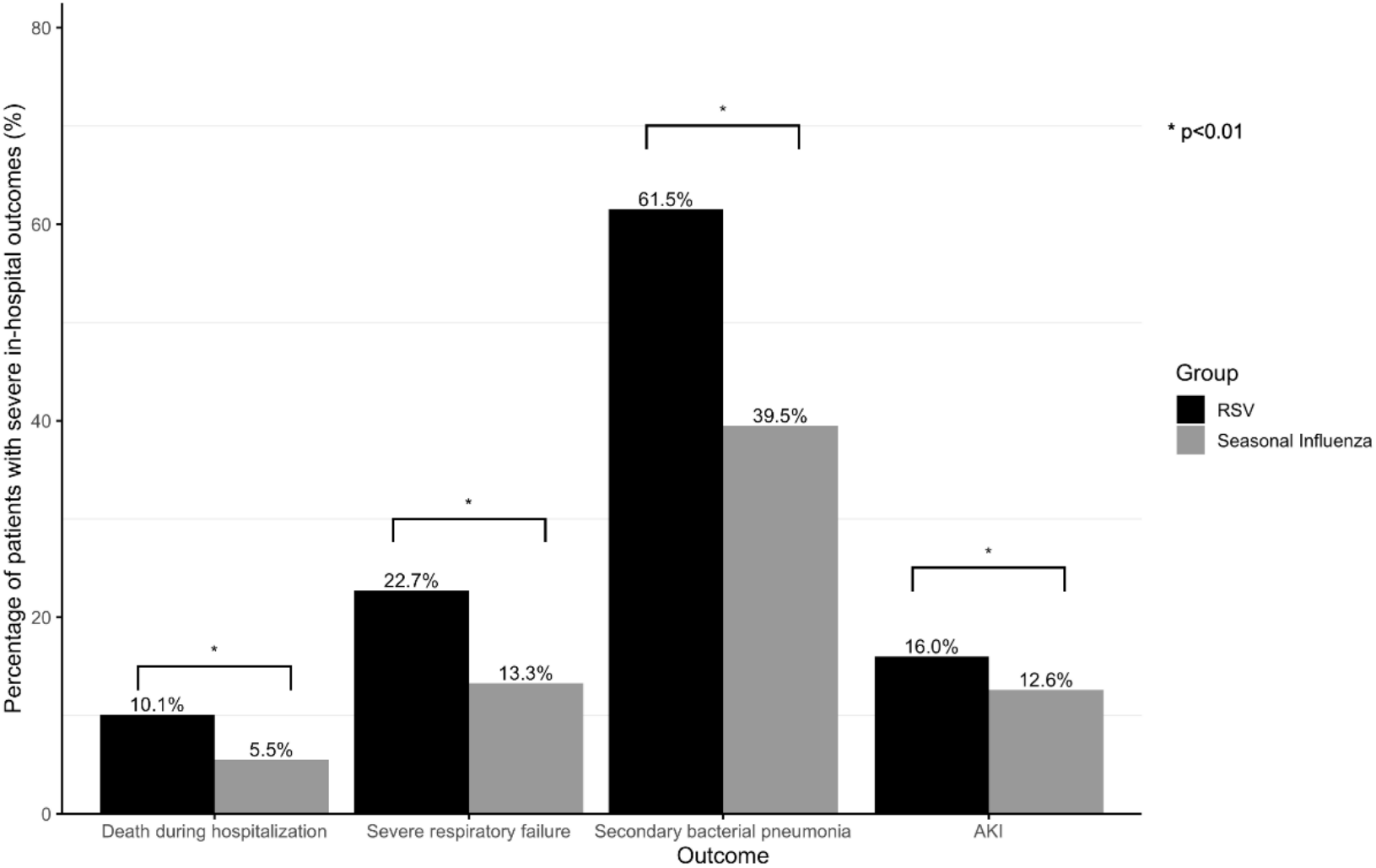
Severe in‐hospital outcomes among influenza and RSV patients in the whole cohort.

**FIGURE 2:**
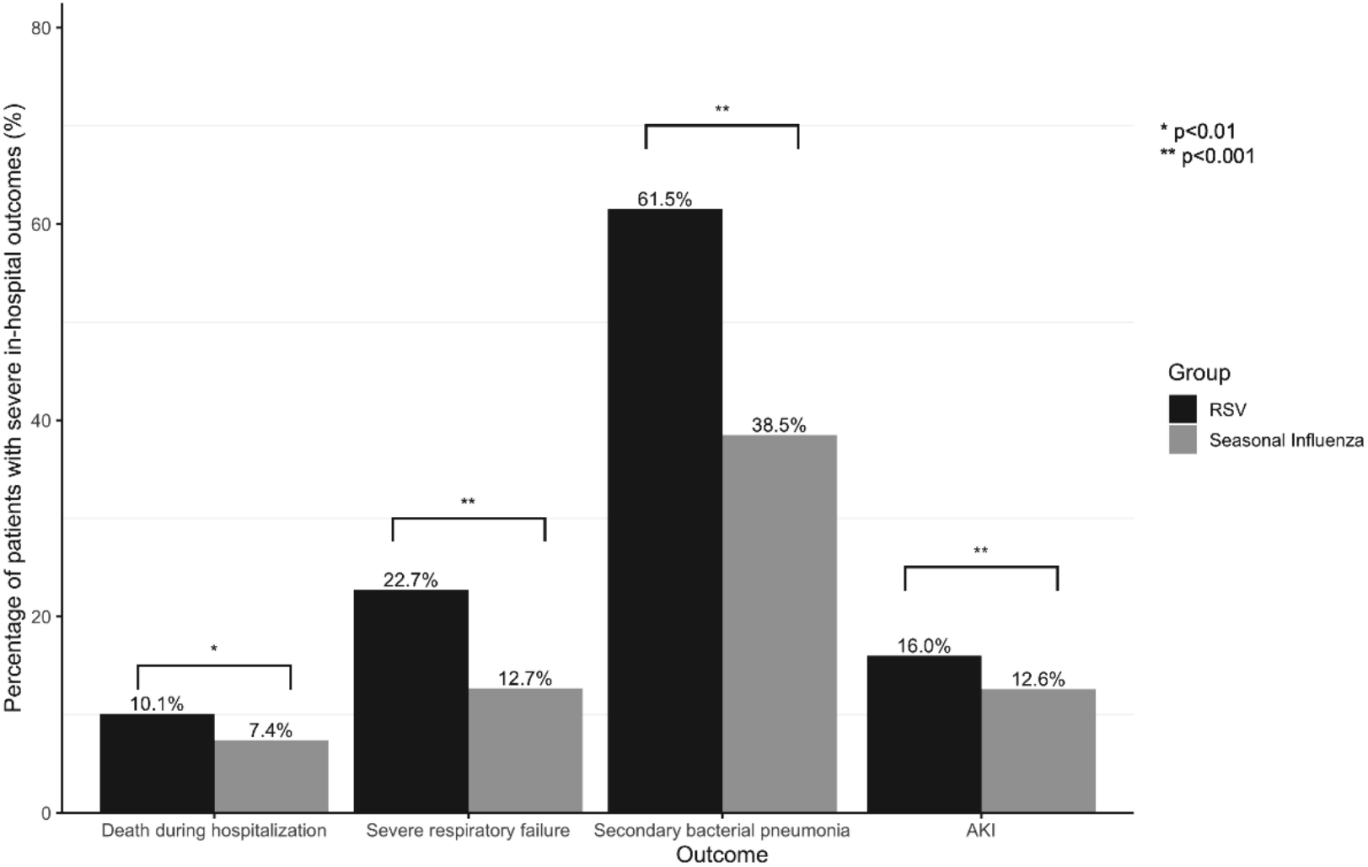
Severe in‐hospital outcomes among influenza and RSV patients in a propensity score matched cohort.

Multivariable analyses demonstrated that RSV infection remained to show a higher risk of death during hospitalization (adjusted odds ratio [aOR] 1.52; 95% CI = 1.13–2.05, *p* < 0.001), SRF (aOR 1.66; 95% CI = 1.43–1.92, *p* < 0.001), secondary bacterial pneumonia (aOR 1.81; 95% CI = 1.61–2.04, *p* < 0.001), and AKI (aOR 1.27; 95% CI = 1.11–1.44, *p* < 0.001) than seasonal influenza (Figure 1 and Table S1). The results remain consistent in analyses using the 1:1 PSM cohort (Figure 2 and Table S2).

The above suggested that patients hospitalized for RSV infection have increased risks for various severe in‐hospital outcomes when compared with patients hospitalized for influenza.

### 3.3 Severe In‐Hospital Outcomes Among Vaccinated and Unvaccinated Influenza Patients, as well as RSV Patients

Among patients hospitalized for influenza infection, those who received influenza vaccines had significantly lower rates of mortality (3.7% vs. 6.2%, *p* < 0.001), SRF (12.0% vs. 16.4%, *p* < 0.001), and secondary bacterial pneumonia (37.9% vs. 43.1%, *p* < 0.001), but not for AKI (16.1% vs. 11.0% *p* < 0.001) compared with the unvaccinated counterparts.

Multivariable analyses demonstrated that patients who received influenza vaccines remained to show a lower risk of death during hospitalization (aOR 0.41; 95% CI = 0.37–0.45, *p* < 0.001), SRF (aOR 0.85; 95% CI = 0.79–0.90, *p* < 0.001), and secondary bacterial pneumonia (aOR 0.71; 95% CI = 0.68–0.75, *p* < 0.001) than those who were unvaccinated, but not for AKI (aOR 0.97, 95% CI = 0.91–1.04, *p* = 0.36).

The incidence and risks of death during hospitalization, SRF, and secondary bacterial pneumonia were significantly higher among RSV patients when compared with unvaccinated influenza patients. The aOR in multivariable analyses was 1.21 (95% CI = 1.07–1.37, *p* = 0.002) for death during hospitalization, 1.45 (95% CI = 1.33–1.59, *p* < 0.001) for SRF, and 1.76 (95% CI = 1.63–1.90, *p* < 0.001) for secondary bacterial pneumonia, when patients with RSV were compared with unvaccinated influenza patients.

The above suggested that patients hospitalized for RSV infection have increased risks for various severe in‐hospital outcomes when compared with patients hospitalized for influenza, regardless of the influenza vaccination status among influenza patients.

#### 3.3.1 Subgroup Analyses

The results in the subgroup analysis in the subgroups aged ≥ 60, < 60, and 50–59 were largely consistent with the results in the full cohort. The results are illustrated in Tables S3–S8. Patients hospitalized for RSV infection, regardless of age, had increased risks for severe in‐hospital outcomes when compared with patients hospitalized for influenza.

### 3.4 Risk Factors for Severe In‐Hospital Outcomes Among RSV Patients

**FIGURE 3:**
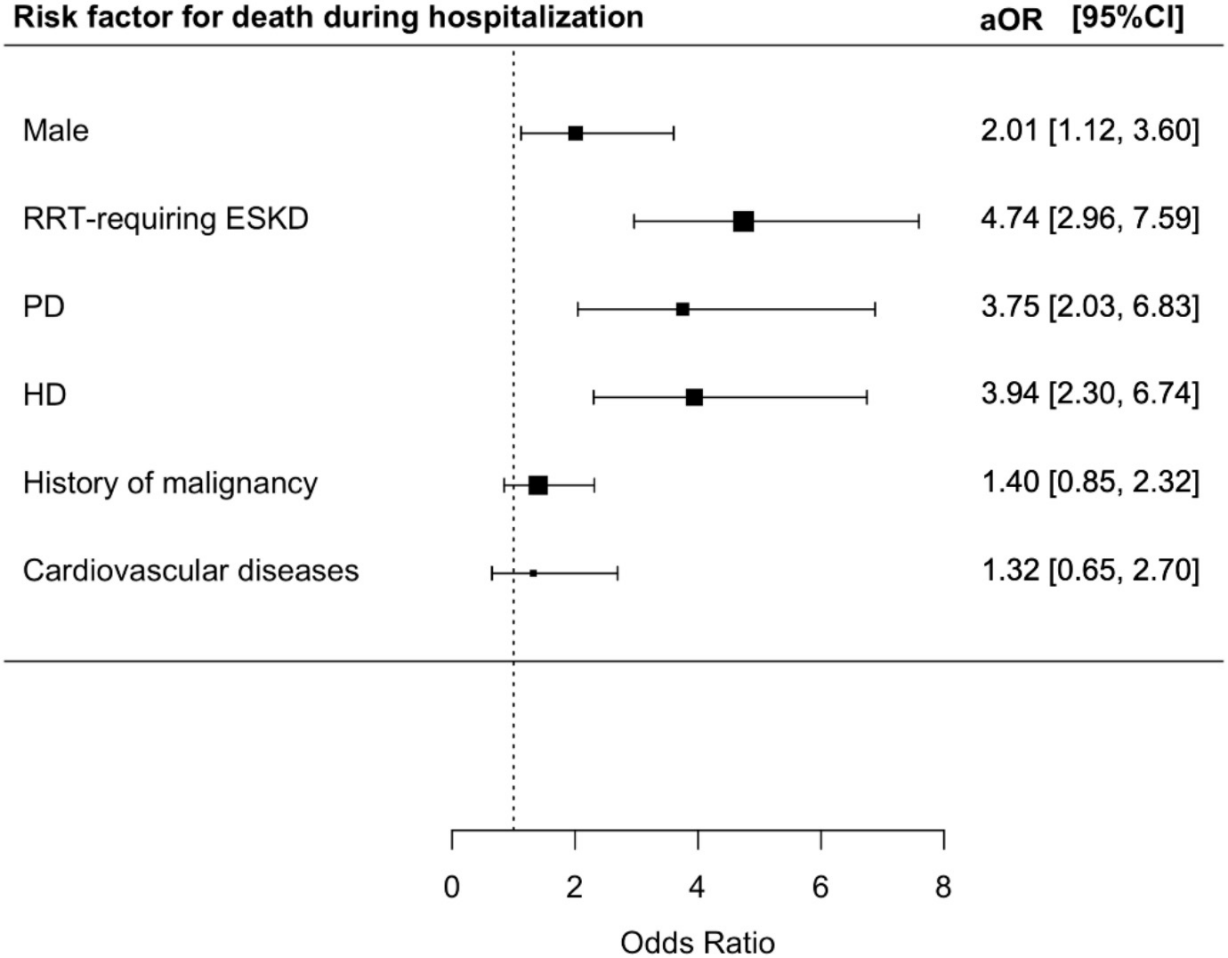
Risk factors for death during hospitalization among RSV patients.

Among adults hospitalized for RSV infection, mortality during hospitalization was increased in male (aOR 2.01; 95% CI = 1.12–3.60, *p* = 0.019), EKSD patients requiring RRT (aOR 4.74; 95% CI = 2.96–7.59, *p* < 0.001), PD (aOR 3.75; 95% CI = 2.03–6.83, *p* < 0.001), and HD (aOR 3.94; 95% CI = 2.30–6.74, *p* < 0.001) (Table S9 and Figure 3).

**FIGURE 4:**
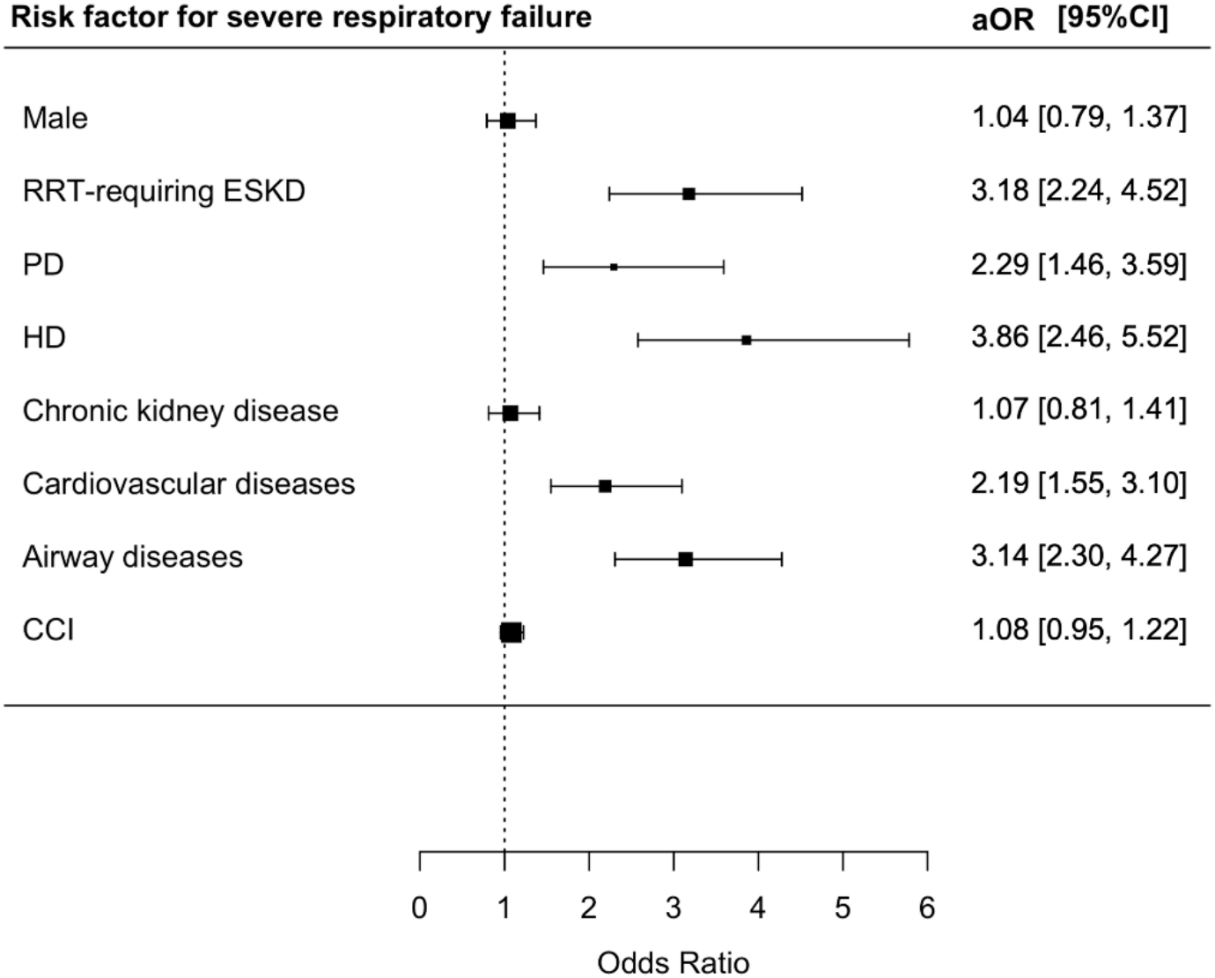
Risk factors for severe respiratory failure among RSV patients.

ESKD requiring RRT (aOR 3.18; 95% CI = 2.24–4.52, *p* < 0.001), PD (aOR 2.29; 95% CI = 1.46–3.59, *p* < 0.001), HD (aOR 3.86; 95% CI = 2.46–5.52, *p* < 0.001), underlying cardiovascular disease (aOR 2.19 95% CI = 1.55–3.10, *p* < 0.001), and airway diseases (aOR 3.14; 95% CI = 2.30–4.27, *p* < 0.001) were independent risk factors for SRF (Table S9 and Figure 4).

**FIGURE 5:**
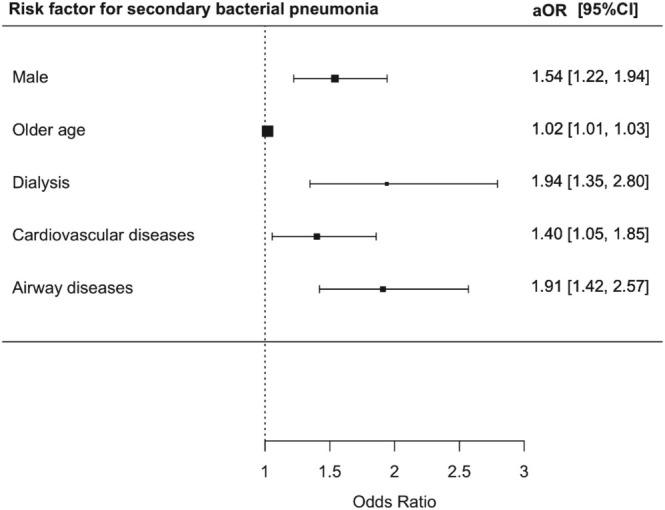
Risk factors for secondary bacterial pneumonia among RSV patients.

Elevated risks of secondary bacterial pneumonia were observed in male (aOR 1.54; 95% CI = 1.22–1.94, *p* < 0.001), older patients (aOR 1.02; 95% CI = 1.01–1.03, *p* = 0.006), patients on dialysis (aOR 1.94; 95% CI = 1.35–2.80, *p* < 0.001), patients with underlying cardiovascular disease (aOR 1.40; 95% CI = 1.05–1.85, *p* = 0.02), and patients with airway diseases (aOR 1.91; 95% CI = 1.42–2.57, *p* < 0.001) (Table S9 and Figure 5). Patients with pre‐existing CKD were predictive of AKI development (aOR 1.58; 95% CI = 1.37–1.81, *p* < 0.001) (Table S9).

#### 3.4.1 Subgroup Analyses

Consistent with the main analyses, subgroup analyses showed that patients hospitalized for RSV infection and received different forms of RRT were associated with increased risk of various in‐hospital outcomes across the different age groups (≥ 60, < 60, and 50–59) (Table S10–S12).

## 4 Discussion

Our study showed that adult patients hospitalized for RSV infection were associated with a significantly higher risk of mortality as well as adverse in‐hospital outcomes than those with seasonal influenza. The findings were seen regardless of influenza vaccination status in influenza patients. The findings are also consistently demonstrated in different age groups, including those aged ≥ 60, < 60, and 50–59, which represented the subgroups that were recommended for different RSV vaccines.

Importantly, ESKD requiring RRT was a robust independent risk factor for unfavorable clinical outcomes in RSV infection. These results call for better strategies to prevent RSV infection, especially vaccination, in vulnerable subjects, irrespective of their age. Patients with cardiopulmonary diseases were also at risk for adverse respiratory outcomes and should also be considered to have vaccination.

Evidence on adults hospitalized for RSV infections largely focuses on older individuals, and data that compare its clinical impact against other respiratory viruses are relatively less available [14, 30, 31, 32]. Our study was by far one of the largest cohorts that compared the clinical outcomes of adults hospitalized for RSV infection against seasonal influenza, in particular with data in the nonelderly populations. We demonstrated that adults hospitalized for RSV infection were associated with a substantially higher risk of in‐hospital mortality compared with seasonal influenza. The results remain robust after adjustment of important confounding factors and also by analysis with the PSM cohort. Although our observation was in line with one recent US study that included 305 RSV cases of adults aged > 60 [9], the underlying mechanisms for such differences in outcomes between RSV and seasonal influenza remain unclear. It is possible that the increased mortality in adults with RSV infection is related to the higher rates of respiratory complications such as SRF and secondary bacterial pneumonia as demonstrated. RSV can lead to lower respiratory tract infections and bronchiolitis [33], which may lead to subsequent respiratory failure. Secondary bacterial pneumonia can occur in over 70% of patients hospitalized for RSV infections, with *Streptococcus pneumoniae* being the most common organism involved [7, 34, 35, 36, 37]. Elderly patients with RSV infection complicated by secondary bacterial pneumonia were reported to have an increase in in‐hospital mortality [36]. Aside from the difference in virulence, it also remains speculative whether the disparity in outcomes may be related to the availability of specific antiviral in seasonal influenza, whereas those with RSV infections only receive supportive therapies. We also postulated that the more favorable outcomes in seasonal influenza may be related to the higher vaccine uptake rates than RSV as the RSV vaccine was only approved in Hong Kong in December 2023 [38]. Such a hypothesis is also supported by one recent study that reported significantly better outcomes of critically ill patients with COVID‐19 infection than those with seasonal influenza infection, of which the discrepancy may also be explained by the marked difference in vaccination rates [39]. Our data also suggested that adults hospitalized for RSV infection had an escalated risk of AKI. These not only affect patient outcomes and well‐being but also confer a burden on healthcare resources. Importantly, our results suggested that nonelderly hospitalized for RSV also showed a significantly increased risk of severe in‐hospital outcomes as elderly, especially in those with underlying comorbidities.

Regarding the risk factors for adverse outcomes among patients with RSV infections, cardiopulmonary diseases were reported in previous studies [40]. In patients with cardiopulmonary diseases, some of the underlying diseases such as COPD or its treatment (inhaled corticosteroid) were well reported to be risk factors for pneumonia. Viral infections, such as RSV infections, can also trigger exacerbation of underlying airway diseases or heart failure. As such, cardiopulmonary disease patients could have increased risks of SRF and secondary bacterial pneumonia as reported in our study. On the other hand, the impact of CKD and ESKD on mortality in RSV infection appeared to be less consistent [41]. In our study, although other comorbidities were also demonstrated to be risk factors for severe in‐hospital outcomes associated with RSV infections and CKD diseases, in particular ESKD requiring RRT, emerged as a strong independent risk factor for severe adverse clinical outcomes among adults hospitalized for RSV infection. In this context, both HD and PD patients showed remarkably high risk for in‐patient mortality and adverse respiratory outcomes in RSV infection. Indeed, ESKD patients show multiple defects in innate and adaptive immunity and thus are highly susceptible to infective complications [42, 43, 44] The findings from our study, in conjunction with the results from other literature, suggested that CKD and ESKD are as important as other medical comorbidities as risk factors for adverse outcomes in RSV infections.

The results of our study have important clinical and public health implications, especially on vaccination policies. Although seasonal influenza vaccines have been widely included in immunization programs in different localities, the recently approved RSV vaccines have received relatively less attention. The current US CDC guidelines recommend RSV vaccines to be given to adults aged ≥ 60 years, but other than that, high‐risk groups are often less well defined because of the paucity of good clinical evidence. Apart from age, the presence of comorbidities is an important consideration when formulating vaccine recommendations. Our present data provide strong evidence on the clinical impact of RSV infection in adults and vulnerable populations and thus call for a revisit on the vaccination recommendations for RSV among adults, especially those with ESKD requiring RRT and other subgroups with comorbidities. In the latest recommendations, RSV vaccinations were also suggested in patients aged below 60 but with risks of severe infection. Adjuvanted subunit vaccine is to be used in persons 50–59 years old, and the bivalent vaccine has been approved for persons 18–59 years of age. In our study, increased severe in‐hospital adverse outcomes were observed in patients below the age of 60 and between 59 and 60, especially those with underlying comorbidities. In this context, it appeared that those with CKD and other comorbidities should be the priority group to receive RSV vaccines. Vaccination in ESKD patients can be a challenging issue as they have shown reduced immunogenicity and efficacy in other vaccines because of impaired immunity [44, 45]. Although RSV vaccines have generally shown good efficacy and safety in older adults, future studies on the immunogenicity and durability of RSV in ESKD patients are required to inform the optimal vaccination strategies in these susceptible individuals. Furthermore, cost‐effectiveness analysis of RSV vaccines in adult patients, particularly those with medical risk factors, is warranted to guide upcoming immunization recommendations. Nonetheless, patients with CKD, especially ESKD, should be recommended for RSV vaccination as in other patients with other medical comorbidities, given the fact that they are at risk of adverse outcomes upon RSV infections.

One point to note is that influenza uptake rate was low in this cohort. For influenza vaccine, various psychological, contextual, sociodemographic, and physical barriers have been postulated [46]. Although RSV vaccines are now commercially available, vaccine hesitance could be an issue. It is important to assess for any barriers to RSV vaccines in the at‐risk population, which can allow policymakers to design a vaccination program, aiming at an increase in RSV vaccine uptake rate.

In this study, we demonstrated how the burden of influenza and RSV infections was affected by the emergence of the COVID‐19 pandemic. It has been well reported that the number of patients with influenza and RSV infections markedly decreased during the COVID‐19 pandemic [13, 15]. Such a phenomenon was also demonstrated in our study. We also noted a resurgence of the cases with influenza and RSV infections in the year 2023, when the various public health policies such as lockdown and social distancing were loosened or removed. During the COVID‐19 pandemic, there were strict public health measures such as universal masking, social distancing, and lockdown. At the same time, all patients diagnosed with COVID‐19, regardless of severity, needed to be hospitalized and isolated until repeated respiratory tract specimens were tested to be repeatedly negative for SARS‐CoV‐2. Some of these measures could offer protection against respiratory viruses other than SARS‐CoV‐2, which can partly account for the reduction of the burden of influenza and RSV infections from 2020 to 2022, whereas the emergence of SARS‐CoV‐2 itself could also contribute to this phenomenon.

Our study has several limitations. First, we have not analyzed the granular details of secondary bacterial infections. Second, data on disease severity scores (e.g., Sequential Organ Failure Assessment [SOFA] Score or APACHE) were not available. Nevertheless, our multivariable regression and PSM cohort have adjusted for most medical comorbidities and disease severity, and the results appeared to be consistent with our main analysis. We have also performed sensitivity analyses to ensure our data is robust across different age groups. There was a small amount of missing data in the ethnicity (< 0.1%), and those subjects with unknown ethnicity were grouped as “others.” Furthermore, our data are derived from a territory‐wide electronic health record system that captures comprehensive clinical information of all adults hospitalized for RSV or seasonal influenza infection during the study period and is therefore a good representation of real‐world data of this clinical entity.

## 5 Conclusions and Policy Implications

Adults hospitalized for RSV infection were associated with a significantly increased risk of in‐patient mortality and adverse outcomes than those with seasonal influenza, and such findings were consistent across different age groups. This calls for an update on RSV vaccination recommendations in adults, especially on the age criteria and considerations of comorbidities.

Given the availability of RSV vaccine as well as the recent approval of vaccine use among patients aged < 59 years old, it is important to consider RSV vaccination among patients who are (1) aged ≥ 60 years and (2) aged < 60 years who are at increased risk for adverse outcomes. The risk factors for severe RSV infections demonstrated in this study provided insights on the target patient subgroups for vaccination, especially among countries and places with limited resources that do not allow vaccination for the entire population.

## Author Contributions

**Wang Chun Kwok:** writing – original draft, writing – review and editing, conceptualization, methodology, data curation, investigation, formal analysis. **Isaac Sze Him Leung:** conceptualization, formal analysis, data curation. **James Chung Man Ho:** conceptualization, writing – original draft, writing – review and editing. **Chung Ki Tsui:** formal analysis, data curation. **David Chi Leung Lam:** writing – original draft, writing – review and editing. **Mary Sau Man Ip:** writing – original draft, writing – review and editing. **Kelvin Kai Wang:** conceptualization, data curation, investigation, methodology, writing – original draft, writing – review and editing, formal analysis. **Desmond Yat Hin Yap:** writing – original draft, writing – review and editing.

## Ethics Statement

The study was approved by the Institutional Review Board (IRB) of the University of Hong Kong and Hospital Authority Hong Kong West Cluster (UW 24‐137). Patient informed consent was waived in this retrospective study by the IRB as it is a retrospective study without active patient recruitment while the data were already deidentified. The study was conducted in compliance with the Declaration of Helsinki.

## Conflicts of Interest

D.Y.H. Yap received research donations from the Wai Im Charitable Foundation, Chan Sui Kau Family Benefits and Charitable Foundation, So Ka Wing and Lee Sau Ying Charitable Foundation, and the Charity Fund from Luk Fook Holdings Limited.

## Patient and Public Involvement Statement

It was not appropriate or possible to involve patients or the public in the design, conduct, reporting, or dissemination plans in this study.

## Supporting information


**Figure S1.** Number of patients hospitalized for influenza and RSV in the study period.
**Table S1.** Severe in‐hospital outcomes among seasonal influenza and RSV patients in whole cohort (aged ≥ 18).
**Table S2.** Severe in‐hospital outcomes among influenza and RSV patients in propensity score matched cohort of age ≥ 18.
**Table S3.** Severe in‐hospital outcomes among seasonal influenza and RSV patients in patients aged ≥ 60.
**Table S4.** Severe in‐hospital outcomes among seasonal influenza and RSV patients in the cohort aged <60.
**Table S5.** Severe in‐hospital outcomes among seasonal influenza and RSV patients in the cohort aged 50–59.
**Table S6.** Severe in‐hospital outcomes among influenza and RSV patients in propensity score matched cohort of age ≥ 60.
**Table S7.** Severe in‐hospital outcomes among influenza and RSV patients in propensity score matched cohort of age < 60.
**Table S8.** Severe in‐hospital outcomes among influenza and RSV patients in propensity score matched cohort of age 50–59.
**Table S9.** Risk factors for severe in‐hospital outcomes among RSV patients in the whole cohort.
**Table S10.** Risk factors for severe in‐hospital outcomes among RSV patients in subgroup aged ≥ 60.
**Table S11.** Risk factors for severe in‐hospital outcomes among RSV patients in subgroup aged < 60.
**Table S12.** Risk factors for severe in‐hospital outcomes among RSV patients in subgroup aged 50–59.

## Data Availability

All available data are presented in the manuscript, and no additional data will be provided. Data are not available to be shared.
