# Supplementary material for: In‐Hospital Mortality and Severe Respiratory and Renal Outcomes—A Territory‐Wide Comparison Between RSV and Influenza
Source: Influenza Other Respir Viruses. 2025 Jun 20;19(6):e70130. doi: 10.1111/irv.70130 (PMC12179560; doi:10.1111/irv.70130)
Supplement: Supplementary file 1 — Figure S1. Number of patients hospitalized for influenza and RSV in the study period. Table S1. Severe in‐hospital outcomes among seasonal influenza and RSV patients in whole cohort (aged ≥ 18). Table S2. Severe in‐hospital outcomes among influenza and RSV patients in propensity score matched cohort of age ≥ 18. Table S3. Severe in‐hospital outcomes among seasonal influenza and RSV patients in patients aged ≥ 60. Table S4. Severe in‐hospital outcomes among seasonal influenza and RSV patients in the cohort aged <60. Table S5. Severe in‐hospital outcomes among seasonal influenza and RSV patients in the cohort aged 50–59. Table S6. Severe in‐hospital outcomes among influenza and RSV patients in propensity score matched cohort of age ≥ 60. Table S7. Severe in‐hospital outcomes among influenza and RSV patients in propensity score matched cohort of age < 60. Table S8. Severe in‐hospital outcomes among influenza and RSV patients in propensity score matched cohort of age 50–59. Table S9. Risk factors for severe in‐hospital outcomes among RSV patients in the whole cohort. Table S10. Risk factors for severe in‐hospital outcomes among RSV patients in subgroup aged ≥ 60. Table S11. Risk factors for severe in‐hospital outcomes among RSV patients in subgroup aged < 60. Table S12. Risk factors for severe in‐hospital outcomes among RSV patients in subgroup aged 50–59. [file IRV-19-e70130-s001.docx]

**Supplementary Figure 1 Number of patients hospitalized for influenza and RSV in the study period**

**Supplementary Table S1 Severe in-hospital outcomes among seasonal influenza and RSV patients in whole cohort (Age ≥ 18)**

| **Outcomes** | **Patient groups** | **Number of subjects (%)** | **Univariate analysis** | | | | | | **Multivariate**^†^ **analysis** | | | | |
| --- | --- | --- | --- | --- | --- | --- | --- | --- | --- | --- | --- | --- | --- |
|  |  |  | **OR** | **95 % CI** | | **p-value** | | | **aOR** | **95 % CI** | **p-value** | | |
| **Death during hospitalization*** | **Seasonal influenza (n = 41206)** | 2251 (5.5%) | -Reference- | | | | | | -Reference- | | | | |
|  | **RSV (n = 3565)** | 359 (10.1%) | 1.94 | 1.72 – 2.18 | | | < 0.001 | | 1.52 | 1.13 – 2.05 | | < 0.001 | |
| **Severe respiratory failure*** | **Seasonal influenza (n = 41206)** | 5496 (13.3%) | -Reference- | | | | | | -Reference- | | | | |
|  | **RSV (n = 3565)** | 810 (22.7%) | 1.91 | | 1.76 – 2.08 | | | < 0.001 | 1.66 | 1.43 – 1.92 | | | p < 0.001 |
| **Secondary bacterial pneumonia *** | **Seasonal influenza (n = 41206)** | 16277 (39.5%) | -Reference- | | | | | | -Reference- | | | | |
|  | **RSV (n = 3565)** | 2194 (61.5%) | 2.45 | | 2.29 – 2.63 | | | < 0.001 | 1.81 | 1.61 – 2.04 | | | < 0.001 |
| **AKI*** | **Seasonal influenza (n = 41206)** | 5172 (12.6%) | -Reference- | | | | | | -Reference- | | | | |
|  | **RSV (n = 3565)** | 570 (16.0%) | 1.33 | | 1.21 – 1.46 | | | < 0.001 | 1.27 | 1.11 – 1.44 | | | < 0.001 |

*: Factors that are statistically significant after adjustment for confounders

^†:^ Adjusted for age; sex; ethnic group; baseline CCI; baseline eGFR presence of DM, chronic airway diseases, cardio-/cerebro-vascular diseases and previous vaccination against influenza and pneumococcal polysaccharide and conjugated vaccine

RSV = Respiratory syncytial virus, CI = Confidence interval, HAP = Hospital acquired pneumonia, AKI = Acute kidney injury

**Supplementary Table S2 Severe in-hospital outcomes among influenza and RSV patients in propensity score matched cohort of age ≥ 18**

| **Outcomes** | **Patient groups** | **Number of subjects (%)** | **Univariate analysis** | | | | | | **Multivariate**^†^ **analysis** | | | | |
| --- | --- | --- | --- | --- | --- | --- | --- | --- | --- | --- | --- | --- | --- |
|  |  |  | **OR** | **95 % CI** | | **p-value** | | | **aOR** | **95 % CI** | **p-value** | | |
| **Death during hospitalization*** | **Seasonal influenza (n = 3565)** | 265 (7.4%) | -Reference- | | | | | | -Reference- | | | | |
|  | **RSV (n = 3565)** | 359 (10.1%) | 1.39 | 1.16 – 1.65 | | | < 0.001 | | 1.81 | 1.18 – 2.76 | | 0.006 | |
| **Severe respiratory failure*** | **Seasonal influenza (n = 3565)** | 453 (12.7%) | -Reference- | | | | | | -Reference- | | | | |
|  | **RSV (n = 3565)** | 810 (22.7%) | 2.02 | | 1.78 – 2.29 | | | < 0.001 | 2.29 | 1.86 – 2.81 | | | p < 0.001 |
| **Secondary bacterial pneumonia *** | **Seasonal influenza (n = 3565)** | 1373 (38.5%) | -Reference- | | | | | | -Reference- | | | | |
|  | **RSV (n = 3565)** | 2194 (61.5%) | 2.56 | | 2.32 – 2.81 | | | < 0.001 | 2.40 | 2.06 – 2.80 | | | < 0.001 |
| **AKI*** | **Seasonal influenza (n = 3565)** | 448 (12.6%) | -Reference- | | | | | | -Reference- | | | | |
|  | **RSV (n = 3565)** | 570 (16.0%) | 1.32 | | 1.16 – 1.61 | | | < 0.001 | 1.75 | 1.48 – 2.07 | | | < 0.001 |

*: Factors that are statistically significant after adjustment for confounders

^†:^ Adjusted for age; sex; ethnic group; baseline CCI; baseline eGFR presence of DM, chronic airway diseases, cardio-/cerebro-vascular diseases and previous vaccination against influenza and pneumococcal polysaccharide and conjugated vaccine

RSV = Respiratory syncytial virus, CI = Confidence interval, HAP = Hospital acquired pneumonia, AKI = Acute kidney injury

**Supplementary Table S3 Severe in-hospital outcomes among seasonal influenza and RSV patients in patients age ≥ 60**

| **Outcomes** | **Patient groups** | **Number of subjects (%)** | **Univariate analysis** | | | | | | **Multivariate**^†^ **analysis** | | | |
| --- | --- | --- | --- | --- | --- | --- | --- | --- | --- | --- | --- | --- |
|  |  |  | **OR** | **95 % CI** | | **p-value** | | | **aOR** | **95 % CI** | **p-value** | |
| **Death during hospitalization*** | **Seasonal influenza (n = 28651)** | 2052 (7.2%) | -Reference- | | | | | | -Reference- | | | |
|  | **RSV (n = 2961)** | 333 (11.2%) | 1.64 | 1.45 – 1.86 | | | < 0.001 | | 1.54 | 1.36 – 1.75 | | < 0.001 |
| **Severe respiratory failure*** | **Seasonal influenza (n = 28651)** | 4539 (15.8%) | -Reference- | | | | | | -Reference- | | | |
|  | **RSV (n = 2961)** | 705 (23.8%) | 1.66 | | 1.52 – 1.82 | | | < 0.001 | 1.51 | 1.37 – 1.66 | | p < 0.001 |
| **Secondary bacterial pneumonia *** | **Seasonal influenza (n = 28651)** | 13834 (48.3%) | -Reference- | | | | | | -Reference- | | | |
|  | **RSV (n = 2961)** | 1937 (65.4%) | 2.03 | | 1.87 – 2.19 | | | < 0.001 | 1.85 | 1.70 – 2.01 | | < 0.001 |
| **AKI*** | **Seasonal influenza (n = 28651)** | 4448 (15.5%) | -Reference- | | | | | | -Reference- | | | |
|  | **RSV (n = 2961)** | 497 (16.8%) | 1.10 | | 0.99 – 1.22 | | | 0.072 | 1.01 | 0.91 – 1.13 | | 0.81 |

*: Factors that are statistically significant after adjustment for confounders

^†:^ Adjusted for age; sex; ethnic group; baseline CCI; baseline eGFR presence of DM, chronic airway diseases, cardio-/cerebro-vascular diseases and previous vaccination against influenza and pneumococcal polysaccharide and conjugated vaccine

RSV = Respiratory syncytial virus, CI = Confidence interval, HAP = Hospital acquired pneumonia, AKI = Acute kidney injury

**Supplementary Table S4 Severe in-hospital outcomes among seasonal influenza and RSV patients in the cohort age <60**

| **Outcomes** | **Patient groups** | **Number of subjects (%)** | **Univariate analysis** | | | | | | **Multivariate**^†^ **analysis** | | | | |
| --- | --- | --- | --- | --- | --- | --- | --- | --- | --- | --- | --- | --- | --- |
|  |  |  | **OR** | **95 % CI** | | **p-value** | | | **aOR** | **95 % CI** | **p-value** | | |
| **Death during hospitalization*** | **Seasonal influenza (n = 12555)** | 199 (1.6%) | -Reference- | | | | | | -Reference- | | | | |
|  | **RSV (n = 604)** | 26 (4.3%) | 2.79 | 1.84 – 4.24 | | | < 0.001 | | 1.90 | 1.22 – 2.95 | | 0.005 | |
| **Severe respiratory failure*** | **Seasonal influenza (n = 12555)** | 957 (7.6%) | -Reference- | | | | | | -Reference- | | | | |
|  | **RSV (n = 604)** | 105 (17.4%) | 2.55 | | 2.05 – 3.18 | | | < 0.001 | 1.54 | 1.33 – 1.79 | | | p < 0.001 |
| **Secondary bacterial pneumonia *** | **Seasonal influenza (n = 12555)** | 2443 (19.5%) | -Reference- | | | | | | -Reference- | | | | |
|  | **RSV (n = 604)** | 257 (42.5%) | 3.07 | | 2.59 – 3.62 | | | < 0.001 | 1.66 | 1.04 – 1.84 | | | < 0.001 |
| **AKI*** | **Seasonal influenza (n = 12555)** | 724 (5.8%) | -Reference- | | | | | | -Reference- | | | | |
|  | **RSV (n = 604)** | 73 (12.1%) | 2.25 | | 1.74 – 2.90 | | | < 0.001 | 1.38 | 1.04 – 1.84 | | | 0.027 |

*: Factors that are statistically significant after adjustment for confounders

^†:^ Adjusted for age; sex; ethnic group; baseline CCI; baseline eGFR presence of DM, chronic airway diseases, cardio-/cerebro-vascular diseases and previous vaccination against influenza and pneumococcal polysaccharide and conjugated vaccine

RSV = Respiratory syncytial virus, CI = Confidence interval, HAP = Hospital acquired pneumonia, AKI = Acute kidney injury

**Supplementary Table S5 Severe in-hospital outcomes among seasonal influenza and RSV patients in the cohort age 50 - 59**

| **Outcomes** | **Patient groups** | **Number of subjects (%)** | **Univariate analysis** | | | | | | **Multivariate**^†^ **analysis** | | | | |
| --- | --- | --- | --- | --- | --- | --- | --- | --- | --- | --- | --- | --- | --- |
|  |  |  | **OR** | **95 % CI** | | **p-value** | | | **aOR** | **95 % CI** | **p-value** | | |
| **Death during hospitalization*** | **Seasonal influenza (n = 4744)** | 146 (3.1%) | -Reference- | | | | | | -Reference- | | | | |
|  | **RSV (n = 307)** | 21 (6.8%) | 2.31 | 1.44 – 3.71 | | | < 0.001 | | 1.94 | 1.18 – 3.20 | | 0.009 | |
| **Severe respiratory failure*** | **Seasonal influenza (n = 4744)** | 554 (11.7%) | -Reference- | | | | | | -Reference- | | | | |
|  | **RSV (n = 307)** | 68 (22.1%) | 2.15 | | 1.62 – 2.86 | | | < 0.001 | 1.56 | 1.13 – 2.13 | | | p < 0.001 |
| **Secondary bacterial pneumonia *** | **Seasonal influenza (n = 4744)** | 1272 (26.8%) | -Reference- | | | | | | -Reference- | | | | |
|  | **RSV (n = 307)** | 148 (48.2%) | 2.54 | | 2.01 – 3.21 | | | < 0.001 | 2.17 | 1.70 – 2.77 | | | < 0.001 |
| **AKI*** | **Seasonal influenza (n = 4744)** | 403 (8.5%) | -Reference- | | | | | | -Reference- | | | | |
|  | **RSV (n = 307)** | 42 (13.7%) | 1.71 | | 1.21 – 2.40 | | | 0.002 | 1.17 | 0.80 – 1.70 | | | 0.42 |

*: Factors that are statistically significant after adjustment for confounders

^†:^ Adjusted for age; sex; ethnic group; baseline CCI; baseline eGFR presence of DM, chronic airway diseases, cardio-/cerebro-vascular diseases and previous vaccination against influenza and pneumococcal polysaccharide and conjugated vaccine

RSV = Respiratory syncytial virus, CI = Confidence interval, HAP = Hospital acquired pneumonia, AKI = Acute kidney injury

**Supplementary Table S6 Severe in-hospital outcomes among influenza and RSV patients in propensity score matched cohort of age ≥ 60**

| **Outcomes** | **Patient groups** | **Number of subjects (%)** | **Univariate analysis** | | | | | | **Multivariate**^†^ **analysis** | | | | |
| --- | --- | --- | --- | --- | --- | --- | --- | --- | --- | --- | --- | --- | --- |
|  |  |  | **OR** | **95 % CI** | | **p-value** | | | **aOR** | **95 % CI** | **p-value** | | |
| **Death during hospitalization*** | **Seasonal influenza (n = 2988)** | 256 (8.6%) | -Reference- | | | | | | -Reference- | | | | |
|  | **RSV (n = 2961)** | 333 (11.2%) | 1.35 | 1.14 – 1.61 | | | < 0.001 | | 1.34 | 1.11 – 1.59 | | 0.001 | |
| **Severe respiratory failure*** | **Seasonal influenza (n = 2988)** | 403 (13.5%) | -Reference- | | | | | | -Reference- | | | | |
|  | **RSV (n = 2961)** | 705 (23.8%) | 2.00 | | 1.75 – 2.29 | | | < 0.001 | 2.07 | 1.80 – 2.38 | | | p < 0.001 |
| **Secondary bacterial pneumonia *** | **Seasonal influenza (n = 2988)** | 1284 (43.0%) | -Reference- | | | | | | -Reference- | | | | |
|  | **RSV (n = 2961)** | 1937 (65.4%) | 2.51 | | 2.26 – 2.79 | | | < 0.001 | 2.70 | 2.42 – 3.01 | | | < 0.001 |
| **AKI*** | **Seasonal influenza (n = 2988)** | 402 (13.5%) | -Reference- | | | | | | -Reference- | | | | |
|  | **RSV (n = 2961)** | 497 (16.8%) | 1.30 | | 1.13 – 1.50 | | | < 0.001 | 1.28 | 1.10 – 1.48 | | | 0.001 |

*: Factors that are statistically significant after adjustment for confounders

^†:^ Adjusted for age; sex; ethnic group; baseline CCI; baseline eGFR presence of DM, chronic airway diseases, cardio-/cerebro-vascular diseases and previous vaccination against influenza and pneumococcal polysaccharide and conjugated vaccine

RSV = Respiratory syncytial virus, CI = Confidence interval, HAP = Hospital acquired pneumonia, AKI = Acute kidney injury

**Supplementary Table S7 Severe in-hospital outcomes among influenza and RSV patients in propensity score matched cohort of age < 60**

| **Outcomes** | **Patient groups** | **Number of subjects (%)** | **Univariate analysis** | | | | | | **Multivariate**^†^ **analysis** | | | | |
| --- | --- | --- | --- | --- | --- | --- | --- | --- | --- | --- | --- | --- | --- |
|  |  |  | **OR** | **95 % CI** | | **p-value** | | | **aOR** | **95 % CI** | **p-value** | | |
| **Death during hospitalization*** | **Seasonal influenza (n = 577)** | 11 (1.9%) | -Reference- | | | | | | -Reference- | | | | |
|  | **RSV (n = 604)** | 26 (4.3%) | 2.32 | 1.13 – 4.73 | | | 0.021 | | 2.20 | 1.05 – 4.64 | | 0.038 | |
| **Severe respiratory failure*** | **Seasonal influenza (n = 577)** | 50 (8.7%) | -Reference- | | | | | | -Reference- | | | | |
|  | **RSV (n = 604)** | 105 (17.4%) | 2.22 | | 1.55 – 3.17 | | | < 0.001 | 1.98 | 1.34 – 2.93 | | | < 0.001 |
| **Secondary bacterial pneumonia *** | **Seasonal influenza (n = 577)** | 89 (15.4%) | -Reference- | | | | | | -Reference- | | | | |
|  | **RSV (n = 604)** | 257 (42.5%) | 4.06 | | 3.08 – 5.36 | | | < 0.001 | 2.93 | 2.06 – 5.31 | | | < 0.001 |
| **AKI** | **Seasonal influenza (n = 577)** | 46 (8.0%) | -Reference- | | | | | | -Reference- | | | | |
|  | **RSV (n = 604)** | 73 (12.1%) | 1.59 | | 1.08 – 2.34 | | | 0.02 | 1.30 | 0.84 – 2.00 | | | 0.24 |

*: Factors that are statistically significant after adjustment for confounders

^†:^ Adjusted for age; sex; ethnic group; baseline CCI; baseline eGFR presence of DM, chronic airway diseases, cardio-/cerebro-vascular diseases and previous vaccination against influenza and pneumococcal polysaccharide and conjugated vaccine

RSV = Respiratory syncytial virus, CI = Confidence interval, HAP = Hospital acquired pneumonia, AKI = Acute kidney injury

**Supplementary Table 8 Severe in-hospital outcomes among influenza and RSV patients in propensity score matched cohort of age 50 - 59**

| **Outcomes** | **Patient groups** | **Number of subjects (%)** | **Univariate analysis** | | | | | | **Multivariate**^†^ **analysis** | | | | |
| --- | --- | --- | --- | --- | --- | --- | --- | --- | --- | --- | --- | --- | --- |
|  |  |  | **OR** | **95 % CI** | | **p-value** | | | **aOR** | **95 % CI** | **p-value** | | |
| **Death during hospitalization*** | **Seasonal influenza (n = 296)** | 17 (2.4%) | -Reference- | | | | | | -Reference- | | | | |
|  | **RSV (n = 307)** | 21 (6.8%) | 3.03 | 1.27 – 7.24 | | | 0.013 | | 2.96 | 1.19 – 7.33 | | 0.019 | |
| **Severe respiratory failure*** | **Seasonal influenza (n = 296)** | 35 (11.8%) | -Reference- | | | | | | -Reference- | | | | |
|  | **RSV (n = 307)** | 68 (22.1%) | 2.12 | | 1.36 – 3.31 | | | < 0.001 | 1.90 | 1.16 – 3.13 | | | 0.011 |
| **Secondary bacterial pneumonia *** | **Seasonal influenza (n = 296)** | 57 (19.3%) | -Reference- | | | | | | -Reference- | | | | |
|  | **RSV (n = 307)** | 148 (48.2%) | 3.90 | | 2.71 – 5.63 | | | < 0.001 | 3.93 | 2.65 – 5.85 | | | < 0.001 |
| **AKI*** | **Seasonal influenza (n = 296)** | 28 (9.5%) | -Reference- | | | | | | -Reference- | | | | |
|  | **RSV (n = 307)** | 42 (13.7%) | 1.52 | | 0.91 – 2.52 | | | 0.11 | 1.21 | 0.67 – 2.15 | | | 0.51 |

*: Factors that are statistically significant after adjustment for confounders

^†:^ Adjusted for age; sex; ethnic group; baseline CCI; baseline eGFR presence of DM, chronic airway diseases, cardio-/cerebro-vascular diseases and previous vaccination against influenza and pneumococcal polysaccharide and conjugated vaccine

RSV = Respiratory syncytial virus, CI = Confidence interval, HAP = Hospital acquired pneumonia, AKI = Acute kidney injury

**Supplementary Table 9 Risk factors for severe in-hospital outcomes among RSV patients in the whole cohort**

|  | **Univariate analysis** | | | **Multivariate analysis**^†^ | | |
| --- | --- | --- | --- | --- | --- | --- |
| **Death during hospitalization** | | | | | | |
|  | **OR** | **95% CI** | **p-value** | **aOR** | **95% CI** | **p-value** |
| **Male*** | 1.43 | 1.15 – 1.78 | 0.001 | 2.01 | 1.12 – 3.60 | 0.019 |
| **ESKD requiring RRT*** | 2.17 | 1.46 – 3.22 | <0.001 | 4.74 | 2.96 – 7.59 | <0.001 |
| **PD*** | 1.78 | 1.03 – 3.07 | 0.039 | 3.75 | 2.03 – 6.83 | <0.001 |
| **HD*** | 2.07 | 1.29 – 3.33 | 0.003 | 3.94 | 2.30 – 6.74 | <0.001 |
| **History of malignancies** | 1.79 | 1.27 – 2.52 | <0.001 | 1.40 | 0.85 – 2.32 | 0.19 |
| **Cardiovascular diseases** | 1.28 | 1.03 – 1.60 | 0.025 | 1.32 | 0.65 – 2.70 | 0.45 |
| **Severe respiratory failure** | | | | | | |
|  | **OR** | **95% CI** | **p-value** | **aOR** | **95% CI** | **p-value** |
| **Male** | 1.33 | 1.14 – 1.55 | 0.001 | 1.04 | 0.79 – 1.37 | 0.79 |
| **ESKD requiring RRT*** | 2.89 | 2.12 – 3.93 | <0.001 | 3.18 | 2.24 – 4.52 | <0.001 |
| **PD*** | 2.21 | 1.46 – 3.35 | 0.039 | 2.29 | 1.46 – 3.59 | <0.001 |
| **HD*** | 3.47 | 2.40 – 5.01 | <0.001 | 3.86 | 2.46 – 5.52 | <0.001 |
| **Chronic kidney disease** | 1.37 | 1.14 – 1.64 | 0.001 | 1.07 | 0.81 – 1.41 | 0.63 |
| **Cardiovascular diseases*** | 1.90 | 1.62 – 2.22 | <0.001 | 2.19 | 1.55 – 3.10 | <0.001 |
| **Airway diseases*** | 3.46 | 2.92 – 4.11 | <0.001 | 3.14 | 2.30 – 4.27 | <0.001 |
| **CCI** | 1.13 | 1.09 – 1.17 | <0.001 | 1.08 | 0.95 – 1.22 | 0.25 |
| **Secondary bacterial pneumonia** | | | | | | |
|  | **OR** | **95% CI** | **p-value** | **aOR** | **95% CI** | **p-value** |
| **Male*** | 1.39 | 1.21 – 1.59 | <0.001 | 1.54 | 1.22 – 1.94 | <0.001 |
| **Older age*** | 1.03 | 1.03 – 1.03 | <0.001 | 1.02 | 1.01 – 1.03 | 0.006 |
| **Dialysis*** | 1.34 | 0.96 – 1.86 | 0.09 | 1.94 | 1.35 – 2.80 | <0.001 |
| **Cardiovascular diseases*** | 1.99 | 1.73 – 2.39 | <0.001 | 1.40 | 1.05 – 1.85 | 0.02 |
| **Airway diseases*** | 1.71 | 1.45 – 2.03 | <0.001 | 1.91 | 1.42 – 2.57 | <0.001 |
| **AKI** | | | | | | |
|  | **OR** | **95% CI** | **p-value** | **aOR** | **95% CI** | **p-value** |
| **CKD*** | 4.31 | 3.56 – 5.21 | <0.001 | 1.58 | 1.37 – 1.81 | <0.001 |
| **Diabetes mellitus** | 1.87 | 1.55 – 2.25 | <0.001 | 0.96 | 0.73 – 1.27 | 0.80 |
| **History of malignancies** | 1.70 | 1.27 – 2.28 | <0.001 | 1.50 | 0.94 – 2.39 | 0.09 |

*: Factors that are statistically significant after adjustment for confounders

^†:^ Adjustment done for confounders including age; sex; ethnic group; baseline CCI; baseline eGFR presence of DM, chronic airway diseases, cardio-/cerebro-vascular diseases and previous vaccination against influenza and pneumococcal polysaccharide and conjugated vaccine

RSV = Respiratory syncytial virus, CI = Confidence interval; HAP = Hospital acquired pneumonia, AKI = Acute kidney injury; PD = Peritoneal dialysis; HD = Haemodialysis; RRT = Renal replacement therapy; CCI = Charlson comorbidity index; CKD = Chronic kidney disease; ESKD = End-stage kidney disease

**Supplementary Table S10 Risk factors for severe in-hospital outcomes among RSV patients in subgroup age ≥ 60**

|  | **Univariate analysis** | | | **Multivariate analysis** | | |
| --- | --- | --- | --- | --- | --- | --- |
| **Death during hospitalization** | | | | | | |
|  | **OR** | **95% CI** | **p-value** | **aOR** | **95% CI** | **p-value** |
| **RRT-requiring ESKD*** | 2.19 | 1.39– 3.46 | <0.001 | 3.81 | 2.32 – 6.27 | <0.001 |
| **HD** | 2.12 | 1.23– 3.66 | 0.01 | 3.39 | 1.90 – 6.05 | <0.001 |
| **Older age** | 1.03 | 1.02 – 1.04 | <0.001 | 1.04 | 1.03 – 1.06 | <0.001 |
| **SRF** | | | | | | |
|  | **OR** | **95% CI** | **p-value** | **aOR** | **95% CI** | **p-value** |
| **RRT-requiring ESKD*** | 2.60 | 1.79 – 3.78 | <0.001 | 2.98 | 1.99 – 4.47 | <0.001 |
| **CAPD*** | 1.88 | 1.10 – 3.20 | 0.02 | 2.19 | 1.24 – 3.86 | 0.007 |
| **HD*** | 3.01 | 1.94 – 4.69 | <0.001 | 3.38 | 2.11 – 5.41 | <0.001 |
| **Cardiovascular diseases*** | 1.75 | 1.47 – 2.08 | <0.001 | 1.76 | 1.47 – 2.12 | <0.001 |
| **Airway diseases*** | 3.63 | 2.03 – 4.36 | <0.001 | 3.84 | 3.17 – 4.65 | <0.001 |
| **HAP** | | | | | | |
|  | **OR** | **95% CI** | **p-value** | **aOR** | **95% CI** | **p-value** |
| **Male*** | 1.35 | 1.16 – 1.58 | <0.001 | 1.49 | 1.26 – 1.76 | <0.001 |
| **Older age*** | 1.04 | 1.03 – 1.05 | <0.001 | 1.04 | 1.03 – 1.05 | <0.001 |
| **Cardiovascular diseases** | 1.66 | 1.43 – 1.93 | <0.001 | 1.60 | 1.36 – 1.88 | <0.001 |
| **Airway diseases*** | 1.66 | 1.38 – 1.99 | <0.001 | 1.65 | 1.36 – 2.00 | <0.001 |
| **AKI** | | | | | | |
|  | **OR** | **95% CI** | **p-value** | **aOR** | **95% CI** | **p-value** |
| **CKD*** | 4.30 | 3.50 – 5.27 | <0.001 | 2.03 | 1.83 – 2.26 | <0.001 |
| **Diabetes mellitus*** | 1.82 | 1.49 – 2.22 | <0.001 | 1.38 | 1.12 – 1.71 | 0.03 |
| **Cardiovascular diseases*** | 1.48 | 1.22 – 1.80 | <0.001 | 1.39 | 1.13 – 1.72 | 0.02 |

*: Factors that are statistically significant after adjustment for confounders

^†:^ Adjustment done for confounders including age; sex; ethnic group; baseline CCI; baseline eGFR presence of DM, chronic airway diseases, cardio-/cerebro-vascular diseases and previous vaccination against influenza and pneumococcal polysaccharide and conjugated vaccine

RSV = Respiratory syncytial virus, CI = Confidence interval; HAP = Hospital acquired pneumonia; AKI = Acute kidney injury; PD = Peritoneal dialysis; HD = Haemodialysis; RRT = Renal replacement therapy; CCI = Charlson comorbidity index; CKD = Chronic kidney disease; ESKD = End-stage kidney disease

**Supplementary Table S11 Risk factors for severe in-hospital outcomes among RSV patients in subgroup age < 60**

|  | **Univariate analysis** | | | **Multivariate analysis** | | |
| --- | --- | --- | --- | --- | --- | --- |
| **Death during hospitalization** | | | | | | |
|  | **OR** | **95% CI** | **p-value** | **aOR** | **95% CI** | **p-value** |
| **RRT-requiring ESKD*** | 4.80 | 1.99 – 11.60 | <0.001 | 3.21 | 1.00 – 10.45 | 0.05 |
| **HD** | 3.93 | 1.39 – 11.09 | 0.01 | 2.03 | 0.59 – 6.95 | 0.26 |
| **Older age** | 1.08 | 1.03 – 1.14 | 0.005 | 1.05 | 0.99 – 1.12 | 0.08 |
| **SRF** | | | | | | |
|  | **OR** | **95% CI** | **p-value** | **aOR** | **95% CI** | **p-value** |
| **RRT-requiring ESKD*** | 4.97 | 2.80 – 8.81 | <0.001 | 3.01 | 1.43 – 6.36 | 0.004 |
| **CAPD** | 3.90 | 1.97 – 7.71 | 0.02 | 2.02 | 0.90 – 3.38 | 0.09 |
| **HD*** | 6.29 | 3.19 – 12.38 | <0.001 | 3.76 | 1.68 – 8.39 | 0.001 |
| **Cardiovascular diseases** | 2.90 | 1.75 – 4.82 | <0.001 | 1.20 | 0.61 – 2.36 | 0.59 |
| **HAP** | | | | | | |
|  | **OR** | **95% CI** | **p-value** | **aOR** | **95% CI** | **p-value** |
| **Male*** | 1.85 | 1.33 – 2.56 | <0.001 | 1.76 | 1.25 – 2.48 | 0.001 |
| **Dialysis*** | 3.81 | 2.08 – 6.98 | <0.001 | 2.11 | 1.02 – 4.37 | 0.044 |
| **Cardiovascular diseases** | 2.14 | 1.35 – 3.39 | 0.001 | 1.01 | 0.56 – 1.82 | 0.97 |
| **AKI** | | | | | | |
|  | **OR** | **95% CI** | **p-value** | **aOR** | **95% CI** | **p-value** |
| **CKD** | 1.97 | 1.49 – 2.59 | <0.001 | 1.33 | 0.55 – 3.23 | 0.53 |
| **Diabetes mellitus** | 1.87 | 1.07 – 3.29 | 0.029 | 0.52 | 0.24 – 1.12 | 0.10 |
| **Cardiovascular diseases** | 3.64 | 2.09 – 6.34 | <0.001 | 1.57 | 0.74 – 3.32 | 0.24 |

*: Factors that are statistically significant after adjustment for confounders

^†:^ Adjustment done for confounders including age; sex; ethnic group; baseline CCI; baseline eGFR presence of DM, chronic airway diseases, cardio-/cerebro-vascular diseases and previous vaccination against influenza and pneumococcal polysaccharide and conjugated vaccine

RSV = Respiratory syncytial virus, CI = Confidence interval; HAP = Hospital acquired pneumonia; AKI = Acute kidney injury; PD = Peritoneal dialysis; HD = Haemodialysis; RRT = Renal replacement therapy; CCI = Charlson comorbidity index; CKD = Chronic kidney disease; ESKD = End-stage kidney disease

**Supplementary Table S12 Risk factors for severe in-hospital outcomes among RSV patients in subgroup age 50 - 59**

|  | **Univariate analysis** | | | **Multivariate analysis** | | |
| --- | --- | --- | --- | --- | --- | --- |
| **Death during hospitalization** | | | | | | |
|  | **OR** | **95% CI** | **p-value** | **aOR** | **95% CI** | **p-value** |
| **RRT-requiring ESKD*** | 5.67 | 2.16 – 14.86 | <0.001 | 5.20 | 1.22 – 22.16 | 0.026 |
| **CAPD*** | 4.39 | 1.45 – 13.28 | 0.009 | 2.97 | 1.05 – 16.93 | 0.042 |
| **HD** | 4.65 | 1.53 – 14.14 | 0.007 | 2.72 | 0.67 – 11.02 | 0.16 |
| **CKD** | 2.71 | 1.07 – 6.86 | 0.036 | 1.50 | 0.34 – 6.71 | 0.59 |
| **SRF** | | | | | | |
|  | **OR** | **95% CI** | **p-value** | **aOR** | **95% CI** | **p-value** |
| **RRT-requiring ESKD*** | 5.81 | 2.81 – 12.02 | <0.001 | 4.44 | 1.70 – 11.62 | 0.002 |
| **CAPD*** | 4.90 | 2.08 – 11.52 | <0.001 | 2.96 | 1.08 – 8.09 | 0.034 |
| **HD*** | 8.17 | 3.29 – 20.27 | <0.001 | 6.19 | 2.12 – 18.09 | <0.001 |
| **CKD*** | 4.37 | 2.38 – 8.02 | <0.001 | 3.42 | 1.25 – 9.39 | 0.017 |
| **Cardiovascular diseases** | 2.49 | 1.37 – 4.52 | 0.003 | 1.20 | 0.54 – 2.66 | 0.66 |
| **Airway diseases*** | 1.83 | 0.87 – 3.88 | 0.11 | 2.30 | 1.02 – 5.21 | 0.045 |
| **HAP** | | | | | | |
|  | **OR** | **95% CI** | **p-value** | **aOR** | **95% CI** | **p-value** |
| **Male*** | 1.65 | 1.05 – 2.59 | <0.001 | 1.69 | 1.05 – 2.73 | 0.032 |
| **RRT-requiring ESKD*** | 6.48 | 2.61 – 16.09 | <0.001 | 4.52 | 1.53 – 13.30 | 0.006 |
| **CAPD*** | 8.60 | 2.51– 29.48 | <0.001 | 4.75 | 1.27 – 17.78 | 0.021 |
| **HD** | 4.27 | 1.54 – 11.80 | 0.005 | 2.40 | 0.76 – 7.62 | 0.14 |
| **CKD** | 2.23 | 1.25 – 3.98 | 0.007 | 1.20 | 0.46 – 3.15 | 0.71 |
| **Cardiovascular diseases** | 2.50 | 1.42 – 4.40 | 0.001 | 1.25 | 0.61 – 2.58 | 0.55 |
|  | **OR** | **95% CI** | **p-value** | **aOR** | **95% CI** | **p-value** |
| **Renal transplantation*** | 60.00 | 3.27 – 1100.64 | 0.006 | 113.44 | 2.77 – 4645.93 | 0.012 |
| **AKI** | | | | | | |
|  | **OR** | **95% CI** | **p-value** | **aOR** | **95% CI** | **p-value** |
| **CKD** | 3.42 | 1.70 – 6.85 | <0.001 | 1.45 | 0.96 – 2.19 | 0.08 |
| **Diabetes mellitus** | 2.23 | 1.15– 4.49 | 0.018 | 1.19 | 0.53 – 2.68 | 0.68 |
| **Cardiovascular diseases*** | 4.22 | 2.13 – 8.35 | <0.001 | 3.44 | 1.57 – 7.54 | 0.002 |

*: Factors that are statistically significant after adjustment for confounders

^†:^ Adjustment done for confounders including age; sex; ethnic group; baseline CCI; baseline eGFR presence of DM, chronic airway diseases, cardio-/cerebro-vascular diseases and previous vaccination against influenza and pneumococcal polysaccharide and conjugated vaccine

RSV = Respiratory syncytial virus, CI = Confidence interval; HAP = Hospital acquired pneumonia; AKI = Acute kidney injury; PD = Peritoneal dialysis; HD = Haemodialysis; RRT = Renal replacement therapy; CCI = Charlson comorbidity index; CKD = Chronic kidney disease; ESKD = End-stage kidney disease
